# Anterior single odontoid screw placement for type II odontoid fractures: our modified surgical technique and initial results in a cohort study of 15 patients

**DOI:** 10.12688/f1000research.9131.2

**Published:** 2016-11-21

**Authors:** Sunil Munakomi, Karuna Tamrakar, Pramod Kumar Chaudhary, Binod Bhattarai

**Affiliations:** 1 Department of Neurosurgery, College of Medical Sciences, Chitwan, Nepal

**Keywords:** Odontoid fracture, screw placement, technique, outcome

## Abstract

**Objective: **Anterior odontoid screw fixation for type II odontoid fracture is the ideal management option. However in the context of unavailability of an O-arm or neuro-navigation and poor images from the available C-arm may be an obstacle to ideal trajectory and placement of the odontoid screw. We herein detail  our surgical technique so as to ensure a correct trajectory and subsequent good fusion in Type II odontoid fractures. This may be advantageous  in clinical set ups lacking state of the art facilities.

**Methods and Results: **In this cohort study we included 15 consecutive patients who underwent anterior odontoid screw placement. We routinely dissect the longus colli to completely visualize the entire width of C3 body. We then perform a median C2-C3 disectomy followed by creating a gutter in the superior end of C3 body. We then guide the Kirchsner (K) wire purchasing adequate anterior cortex of C2. Rest of the procedure follows the similar steps as described for odontoid screw placement.

We achieved 100% correct trajectory and screw placement in our study. There were no instances of screw break out, pull out or nonunion. There was one patient mortality following myocardial infarction in our study.

**Conclusion: **Preoperative imaging details, proper patient positioning, meticulous dissection, thorough anatomical knowledge and few added surgical nuances are the cornerstones in ideal odontoid screw placement. This may be pivotal in managing  patients in developing nations having rudimentary neurosurgical set up.

## Introduction

Management of type II odontoid fractures has been long debated ^1^. Conservative management, a regular practice in earlier days, was later followed by prolonged application of halo vest. These techniques invariably lead to non union of the fracture and furthermore caused major discomfort to the patients ^1, 2^. It was Nakanishi and Bohler who initially described odontoid screw placement for type II odontoid fractures ^3^. With recent advancements in neurosurgery and additions to its armamentarium with tools like Neuro-navigation and O arm, odontoid screws can now be placed with high accuracy, ease and low morbidity ^4– 6^.

However, in major developing countries like Nepal we still invariably lack these tools, and therefore free hand technique is still the only viable option for the management of such cases. Herein we discuss a simple technique for anterior odontoid screw placement which is comparable to placement of the same under guidance of an 'O' arm or neuro-navigation, in terms of accuracy of the placement, associated complications and peri-operative morbidity to the patients.

## Materials and methods

We included 15 patients from a cohort group in our study who were managed with anterior single odontoid screw placement from 2011–2015 in the Department of Neurosurgery, College of Medical Sciences, Nepal. All the patients were first evaluated with the help of X-ray, computerised tomography (CT) and magnetic resonance imaging (MRI) of the spine. CT was performed to diagnose the type and pattern of the fracture and also to rule out other associated bony injuries. MRI was performed to determine the integrity of the transverse ligament, associated soft tissue injuries and to rule out cord contusions. The disease process was explained, the procedure and the alternate methods of management were thoroughly detailed to all the patients and their family members. Written consent for the management was obtained from all the patients in the inclusion cohort. The study was approved by the ethical board of the College of Medical Sciences, Chitwan, Nepal Patient details including age, sex, mode of injury, neurological grade at presentation (Frankel grading), associated injuries, any peri-operative untoward events and complications related to the procedure were recorded. We used cannulated and partially threaded lag screws from the Medtronic implant system.

Following the procedure we encouraged early mobilization of the patients on a cervical collar from post operative day 2, after performing a CT spine scan to assess the trajectory and location of the screw. We advocated performing a dynamic X-ray cervical spine (lateral view) 4 weeks after the surgery to rule out any evidence of pseudo-arthosis (anterior translation or angulation in the fracture site) or any instances of implant failure. Patients were then advised for follow up visits at the 3 ^rd^, 6 ^th^ and 12 ^th^ month in our spine clinic. Inclusion and exclusion criteria are outlined in Box 1 and Box 2, respectively.

Box 1. **Inclusion criteria**

1. Type 2 transverse fracture.
2. Posterior oblique fracture.
3. Informed consent.

Box 2. **Exclusion criteria**

1. Disrupted transverse ligament (Anterior dens interval (ADI) >4mm).
2. Associated Jefferson’s fracture (overhang of lateral masses of C1 on C2 >7mm)
3. Oblique anterior fracture.
4. Severe osteopenia.
5. Old fractures.
6. Short neck, excessive cervical kyphosis, concomitant thoracic kyphosis and barrel shaped chest.
7. Failure to obtain consent for the procedure.

## Surgical modifications for the procedure

We followed a few modifications to the routine surgical steps in the placement of the odontoid screw. The most common complication of the procedure is the wrong trajectory of the screws that predisposes the patient to early implant break out or pull out and fracture pseudo-arthosis. To ensure this is avoided even in the context of rural set ups lacking an O-arm and navigation facilities, we followed these additional steps during the procedure:

1. Midline trajectory of the screw – For correct positioning of the patient to ensure correct trajectory of the screw in the midline, we ensured that the tip of the nose, supra-sternal notch and the xiphisternum were in the same anatomical line. The head of the patient was then securely fixed to the table with adhesive tape. We routinely then exposed the entire breath of the C3 body by dissecting off the longus colli muscles on either side and marked the midpoint as an anatomical landmark to ensure the midline trajectory. The C-arm images in the antero-posterior (AP) view usually ensure the correct location of the dens in most circumstances. However, the quality of the C-arm and body habitus of the patient may be a major limiting issue in obtaining quality images. This method also obviates the continuous use of a C-arm to take the AP view to ensure its midline trajectory. Ideally biplanar fluoroscopy is required to obtain images in sagital and coronal views. After initial confirmation of the correct pathway, the C-arm can be used for lateral images to ensure its correct crossover of the fracture line, all threads migrating beyond the fracture line and ideal placement of its tip just beneath the cortex of odontoid tip. This minimizes the operating time without compromising on the screw trajectory.

2. Adequate banking of anterior C2 corticol bone support – To limit the issues of early implant break out, we created a small gutter in the superior aspect of C3 body following a median C2–C3 disectomy. Doing so the endplate of C2 can be breached from a more posterior aspect thereby ensuring good anterior cortical support from C2 to the screw.

3. Normal alignment of the fracture segments – In order to prevent non-anatomic fusion, we have classified the fracture of the type II odontoid into anterior, neutral and the posterior variants depending upon the anatomical position of the distal odontoid segment. We then performed controlled neck movements to either flex or extend the neck to bring back the normal alignment between the fracture segments. The use of neuro-physiological studies like SSEP may help us in the process to minimize any inadvertent neurological compromise during the neck manipulation.

4. Post operative morbidity due to screw head positioning – There will be discomfort and sometimes dysphagia owing to the presence of screw head at the C2–C3 inter-space. The gutter we create at the C3 will ideally act as a station for the lodgment of the screw head during neck movements thereby limiting its pressure effect to the anteriorly located trachea-esophageal complex.

## Operative technique

We lack an 'O' arm and navigation system to aid us in ideal placement of odontoid screws. But we believe that detailed analysis of pre-operative radio-images, proper patient positioning and correct operative exposure of anatomical details followed by controlled intra-operative manipulation of the neck help us ensure ideal placement of the odontoid screw.

### Positioning

We routinely placed the patient in supine position with placement of a pad beneath the inter-scapular region to extend the neck so as to maintain the normal cervical lordosis.

### Incision

We used a transverse incision for the medial boarder of the sternocleidomastoid muscle to the midline based on C5–C6 level on the right side. Dissection then proceeds in a similar fashion as compared to the anterior cervical disectomy procedure ^7^.

### Exposure

We routinely exposed the entire breadth of the C3 vertebral body. Longus colli on both sides were dissected off the C3 vertebral body until a clear view of the lateral boarder of C3 is seen on both sides. This is very important as the screw must be placed exactly on the midline. Then with the help of a curette we carried out a C2–3 disectomy on the midline. After partial disectomy, we drilled (or curetted) so as to make a gutter on the superior aspect of the C3 body with depth facing upward. This is very helpful for accurate placement of the odontoid screw behind the anterior cortex of C2 body without deviation from midline. The groove also provides the proper shelter for the screw head.

### Neck manipulation

For simplicity we classified odontoid type II fractures into three types:

1. Type A- Anterior displacement of dens
2. Type B- Neutral
3. Type C- Posterior displacement of dens

**Figure 1.**
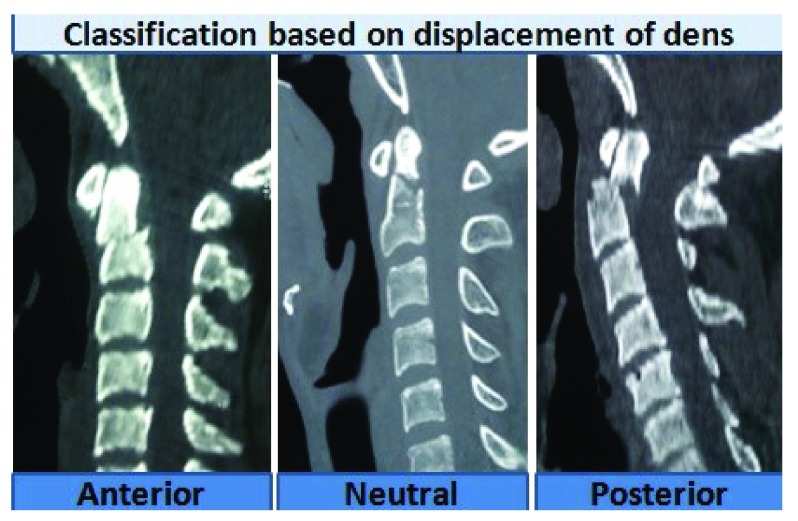
Classification of type II odontoid fractures based on the displacement of distal fracture segment.

This is illustrated in Figure 1.

For type A fracture- we hyperextend the neck as the screw is about to pass the fracture line.

For type B fracture- no neck manipulation is required.

For type C fracture -we flex the neck as the screw is about to enter the fractured line.

### Use of 'C' arm

We regularly do lateral and AP view of the upper cervical spine after positioning of the patient to make sure of normal cervical lordosis and fixed the head with plaster. Lateral view is required initially as we place the 'K' wire on the C2 base. One should ensure the projection of the 'K' wire to be posterior to the anterior cortical layer of C2 to avoid screw break out.

**Figure 2.**
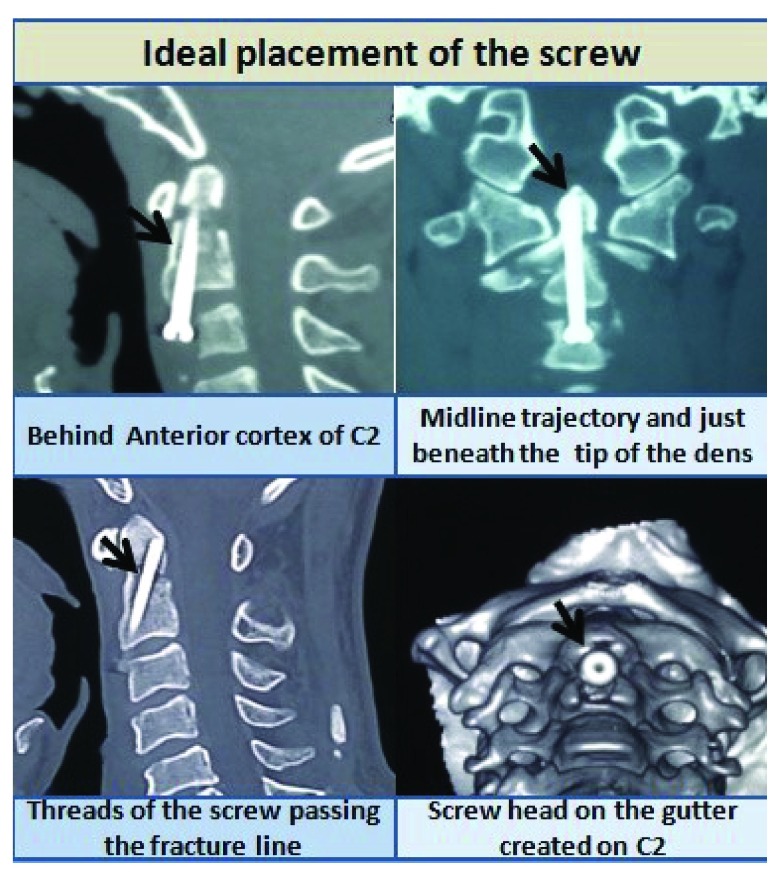
Ideal trajectory, projection and placement of the odontoid screw.

**Figure 3.**
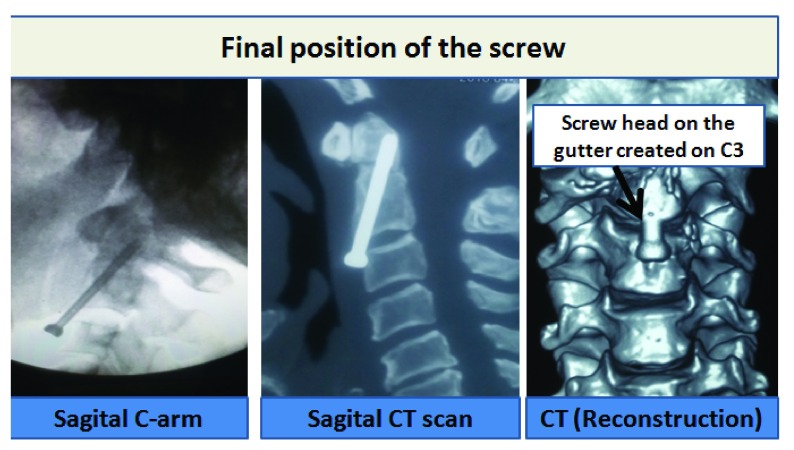
Final position of the odontoid screw after its placement.

After the 'K' wire penetrates the endplate of C2, the 'C' arm is changed for AP views to confirm midline entry of the 'K' wire into the body of the C2 and dens. The ideal trajectory and the final position of the screw following the procedure have been detailed in Figure 2 and Figure 3.

## Results

### Demographic study

In our cohort study, there was a male preponderance (male: female ratio of 6.5: 1). Age of the patients ranged from 15 to 60 years.

### Cause of injury

Road traffic accident was the most common mode of injury in 9 patients (60%) followed by fall injuries in 4 of them (26.67%).

### Associated injuries

There were polytraumas associated with the condition in 9 patients ( 60%).The presence of associated cord contusion was evident in 4 of them (26.67%).

### Clinical presentation

Most of the patients were in Frankel grade E status at presentation (80%). Two patients (13.34%) of the group were in Frankel grade C status and 1 (6.67%) was in Frankel status. The clinical profile of all the patients in the study has been summarized in Table 1.

**Table 1.** Clinical profile of all the patients in the cohort study.

S.No	Age/Sex	Mode of injury	Medical Comorbidities	Symptoms	ASIA grading	Associated injuries
1	34/F	RTA	None	Neck pain	E	None
2	30/M	RTA	None	Neck pain	E	Fracture 3 ^rd^ metacarpal bone
3	22/M	RTA	None	UL weakness	C	C4–C5 cord contusion
4	21/M	Fall injury	None	Neck pain	E	None
5	34/M	RTA	None	UL weakness	C	C2–C3 cord contusion
6	15/M	RTA	None	Neck pain	E	None
7	45/M	RTA	None	Neck pain	E	Lung contusion
8	45/M	RTA	None	Neck pain	E	Left Fronto-temporal SDH
9	28/M	RTA	None	Neck pain	E	None
10	45/M	RTA	Diabetes	Neck pain	E	Bladder rupture
11	40/M	Earthquake	None	Neck pain	E	None
12	60/M	Fall injury	Hypertension	Quadriplegia	A	High cord contusion
13	30/M	Fall injury	None	UL weakness	E	C1–C4 cord contusion
14	31/M	Gas Explosion	None	Neck pain	E	Left femur inter-trochanteric fracture
15	55/M	Fall injury	Hypertension	Neck pain	E	None

*RTA-Road traffic accident/UL-Upper limb/ASIA-American Spinal Injury Association

### Outcome in the patients

In our cohort study, 14 out of 15 cases had excellent post operative outcome. Two of the cases who initially presented with Frankel grade C status on admission had associated cord contusion, with no other evidence of fracture and associated instability. Post operatively, both of them improved to Frankel Grade E.

There were no instances of wrong trajectory or false location of the screw head in our study. During the follow up visits, we found good union of all the fractures without any reports of screw malfunctioning. None of our cases had to be re-operated because of screw related problems or pseudo-arthosis.

Mild discomfort during swallowing was present in 2 cases (13.33%) that improved within few days of the procedure.

We did not have any wound related complications.

In our study group, we had a single mortality following inferior wall myocardial infarction in a 60-year-old male that presented with Frankel grade A neurological status and had associated high cord contusion.

## Discussion

Odontoid type II fracture warrants surgical fixation. Though conservative management with halo rest is an option and still is used in some centers, surgical management is comparatively far more superior with regards to union at the fracture site ^8, 9^.

Development in neurosurgical field has evolved tremendously in recent years Newer armamentarium like neuro-navigation and 'O' arm techniques have now revolutionized complicated surgeries that require a high degree of accuracy and precisions ^4– 6^. In developing countries like ours, despite these intra-operative aids, the procedure can still be performed using pre-operative images and pertaining to our basic anatomical knowledge. Our results are comparable to previously published studies ^10^.

The major advantages of anterior screw fixation are immediate spinal stability with preserved C1–C2 rotation. It also provides high union rate ^11^ The threads at the end of the screw help to couple the fractured segments together (theory behind lag compression) thereby promoting early fusion. There is also no need for autologous bone graft harvesting.

Major limitations of the procedure are the need for intact integrity of the transverse ligament and the prerequisite of attaining normal alignment of the spine before screw placement.

In all of our cases we only used one screw but still attained satisfactory union of the fracture. With our method of complete exposure of the C3 vertebral body, we are able to drill a midline groove on the C3 body which helps us to project the 'K' wire into the dens with good C2 body cortical purchase thereby minimizing the risk of screw break out. Our next technical nuance is the concept of controlled neck manipulation just prior to the 'K' wire entry into the fractured site. This maintains cervical lordosis as well as decreases the chance of dislodgement of the fractured segments and subsequent non-anatomic fusions. With our surgical technique we haven't met any instances of displacement of the fractured segment or need for multiple screws.

We believe multiple screws increase the risk of displacement of fracture segments. Double screws also increase the odds of intra operative failure and surgical difficulties. Moreover, there are no differences in terms of load bearing capacity of the screws as well as the subsequent fusion rate following single or double odontoid screws ^12– 14^.

Anterior odontoid screw placement is a demanding procedure which can invariably lead to major complications. Most of these are related to implant malpositioning and failures. In one study, the procedure had to be abandoned in two cases and there was screw loosening in two patients ^15^. There are also reports of critical neurovascular compromise and severe dysphagia following same procedures ^16– 18^. We did not have such complications in our cohort study.

We achieved 100% fusion rate. The union rate following odontoid screw fixation ranges from 81–100% in the literature ^16^.

Road traffic accidents were the major cause of the injury in our study group (60%) comparable to 80% of case in one recent study ^16^.

The major advantages of our technique are a short learning curve to master it and the execution of the steps even with the use of a single C-arm during the procedure. Another benefit is the decreased operative time with reduced exposure to radiation owing to reduced use of the C-arm for obtaining coronal images.

A major limitation of the study is the small size of our cohort study group. Moreover, anterior approach is not justified in scenarios such as associated transverse ligament disruption, comminuted and significantly displaced fracture segments as well as posterior element injuries ^19^. There is also high incidence of non-union in age groups of above 70 years with concomitant osteoporosis ^20^. Posterior fusion is rather a more valid approach in such cases ^19^.

Whether similar results can be extrapolated to major subsets of other patients remains to be answered. Learning time can be minimized by mastering the technique through cadaveric courses.

We believe that our surgical technique will certainly be a boon in managing patients with odontoid fracture with high therapeutic success and minimal morbidities, especially in the developing regions.

## Conclusion

Most odontoid type II fractures warrant surgical fixation and with proper utilization of our technique, such challenging cases can be conquered with great success. This is even more valid in the context of developing nations where newer tools to aid the procedure are not always available. The benefits of our technique can be summarized as:

1. Alignment of the anatomical landmarks during positioning of the patients and liberal exposure of the width of the C3 body helps us to mark the midline trajectory. This minimizes use of C-arm for obtaining coronal images thereby reducing radiation exposure as well as the operative time.
2. Controlled neck manipulation restores the cervical lordosis and realigns the fracture segments thereby promoting anatomic fusion.
3. Gutter on the C3 body following C2–C3 median disectomy provides corridor for adequate purchase of anterior cortex of C2 thereby minimizing risk of early screw break out. It also stations the head of the screw minimizing pressure to the trachea-oesophageal complex.
