# Supplementary material for: Anterior single odontoid screw placement for type II odontoid fractures: our modified surgical technique and initial results in a cohort study of 15 patients
Source: F1000Res. 2016 Nov 21;5:1681. Originally published 2016 Jul 13. [Version 2] doi: 10.12688/f1000research.9131.2 (PMC5133680; doi:10.12688/f1000research.9131.2)
Supplement: Peer review history

# Peer review: Referee response for version 2

**DOI:** 10.5256/f1000research.11047.r17854

**Published:** 2016-11-29

**Authors:** Yad Ram Yadav

**Affiliations:** 1 Department of Neurosurgery, Netaji Subhash Chandra Bose Medical College, Jabalpur, Madhya Pradesh, India

Good article, revision addressed nicely questions raised in original version. A small correction needs to be made: in Clinical Presentations, the authors did not mention the Frankel grade of one patient. Please correct that.

I have read this submission. I believe that I have an appropriate level of expertise to confirm that it is of an acceptable scientific standard.

# Peer review: Referee response for version 1

**DOI:** 10.5256/f1000research.9825.r16589

**Published:** 2016-09-26

**Authors:** Mohan Raj Sharma, Amit Pradhang

**Affiliations:** 1 Department of Neurosurgery, Institute of Medicine, Tribhuvan University Teaching Hospital, Kathmandu, Nepal

We congratulate the authors for their work on a relatively difficult surgery with modification in a resource constrained setting such as Nepal. However, there are some points which warrant clarification:

**1. Exclusion Criteria**

 Some redundancy is noted in points 1 and 2. Disrupted transverse ligament could mean either Anterior dens interval (ADI) >4mm or in case of Jefferson’s fracture, overhanging of lateral masses of C1 on C2 of >7mm. Our suggestion is to divide them as:

1. Disrupted transverse ligament (Anterior dens interval (ADI) >4mm)
2. Associated Jefferson’s fracture (Overhang of lateral masses of C1 on C2 >7mm)

**2. Surgical modifications for the procedure**

*Midline trajectory of the screw- The C-arm images in the antero-posterior (AP) view usually ensures the correct alignment of the k-wire with the dens.*  It is not clear whether the authors routinely used the biplanar fluoroscopy or AP view was sufficient.

**3. Clinical presentation**

 Frankel grade of the last patienthas not been mentioned. We would have used the ASIA grading which is a better and universally accepted grading system for impairment following spinal cord injury.

 Overall, though this is a small serious with one year follow up, this will definitely stimulate other neuro and spine surgeons in Nepal to carry out such study in future.

 Dr. Mohan R. Sharma, MS, Dr. Amit Pradhang, MS, MCh

We have read this submission. We believe that we have an appropriate level of expertise to confirm that it is of an acceptable scientific standard.

# Peer review: Referee response for version 1

**DOI:** 10.5256/f1000research.9825.r15625

**Published:** 2016-09-01

**Authors:** Yad Ram Yadav

**Affiliations:** 1 Department of Neurosurgery, Netaji Subhash Chandra Bose Medical College, Jabalpur, Madhya Pradesh, India

Although there was no elderly patient in present series, is this technique enough for geriatic patients (osteoporosis and atlantoaxial arthritis)? Do any one have to use anything to augment screw fixation in osteoporotic fracture.

 Authors should also mention risk factors (in whom it is likely to fail).

I have read this submission. I believe that I have an appropriate level of expertise to confirm that it is of an acceptable scientific standard, however I have significant reservations, as outlined above.
